# Barriers to accessing contraceptive services for adolescents in Sub-Saharan Africa: a scoping review

**DOI:** 10.1080/16549716.2026.2699036

**Published:** 2026-07-13

**Authors:** Pascal Kingsley Mwin, Saeed Jabactey Abdullah, Ona McCarthy, Vera-Irene Kokoi, Rashida Ferrand

**Affiliations:** a Department of Non-Communicable Disease, World Health Organisation, Accra, Ghana; b Department of Food Safety, Food and Drugs Authority, Accra, Ghana; c Department of Epidemiology, London School of Hygiene & Tropical Medicine, London, UK; d School of Public Health, University of Ghana, Accra, Ghana

**Keywords:** Young people, low- and middle-income countries, sexual and reproductive health, contraceptive uptake, family planning services

## Abstract

Adolescents in Sub-Saharan Africa (SSA) continue to face significant barriers to accessing contraceptive services, despite global and regional commitments to improve sexual and reproductive health (SRH) outcomes. This scoping review synthesises current evidence on the barriers adolescents encounter when seeking contraceptive services across SSA. Following the Arksey and O’Malley framework and the Joanna Briggs Institute guidelines, a comprehensive literature search of peer-reviewed and grey literature published between January 2010 and January 2025 across multiple databases was conducted. A total of 30 studies met the inclusion criteria. Thematic synthesis revealed five major categories of barriers: individual-level [limited knowledge, fear of side effects, misconceptions], sociocultural [stigma, religious beliefs, parental disapproval], economic [cost of services and transportation], health system-related [provider attitudes, confidentiality concerns, facility accessibility], and policy/legal [age restrictions, mandatory parental consent, poor coordination among agencies]. The findings highlight how these multi-level barriers intersect to impede adolescents’ access to contraception, contributing to high rates of early pregnancy, unsafe abortion, and sexually transmitted infections. Notably, research gaps remain regarding marginalised groups such as LGBTQ+ youth and adolescents with disabilities. The review emphasises the need for adolescent-centred policy reforms, improved provider training, expanded community engagement, and equitable financing mechanisms to support adolescent access to contraception. These findings can inform strategies for achieving the Sustainable Development Goals (SDGs) related to health and gender equality in Sub-Saharan Africa.

## Paper Context

**Main findings:** There exist persistent individual, sociocultural, economic, policy and legal restrictions, and health system barriers that reinforce adolescents’ resistance to accessing contraceptive services in Sub-Saharan Africa.

**Added knowledge:** This review provides consolidated evidence on the barriers that reinforce adolescents’ resistance to accessing contraceptive services in Sub-Saharan Africa, by providing a structured overview to guide research, practice, and policy discussions.

**Global health impact for policy and action:** By consolidating current knowledge, the review guides global health strategies and policy actions to strengthen adolescent-centred contraceptive service delivery and advance sexual and reproductive health equity across low-resource settings.

## Background

Global efforts to improve sexual and reproductive health (SRH) outcomes have witnessed significant progress over recent decades, supported by national and international commitments, particularly the Sustainable Development Goals (SDGs) (2015–2030) [1]. In particular, SDG Target 3.7 promotes universal access to SRH services, while Target 5.6 focuses on reproductive rights in line with the International Conference on Population and Development (ICPD) (1994) and the Beijing Platform for Action (1995), a landmark global agenda for gender equality and women’s empowerment [2–4].

Adolescence [10–19 years], as defined by the World Health Organisation (WHO), is a formative period marked by unique reproductive health needs [5,6]. Yet adolescents in Sub-Saharan Africa (SSA) face the highest prevalence of unmet contraceptive needs globally, with nearly one in four women of reproductive age in the region lacking access to modern contraceptive methods [7,8]. This gap contributes to staggering health outcomes: SSA reports some of the highest adolescent birth rates in the world, exceeding 200 per 1,000 girls in countries like Niger, and approximately 13% of pregnancies in the region end in abortion, 97% of which are unsafe [9]. These challenges not only pose a major threat to maternal health but also undermine progress toward broader goals of health equity and gender equality [10].

While it is recognised that adolescents in this region encounter sociocultural, legal, and economic obstacles, existing evidence remains fragmented and often limited to single-country studies. There is a notable lack of regional synthesis that analyses how these multi-level barriers intersect to impede access. This review addresses this gap by applying the Socio-Ecological Model (SEM) to provide a structured, comprehensive mapping of barriers at the individual, interpersonal, community, and structural levels [11–13]. By synthesising evidence from both peer-reviewed and grey literature over the last 15 years, this study offers a new regional perspective intended to inform more targeted, adolescent-responsive interventions and policy reforms across SSA.

The review focuses on the period from 2010 to 2025, a timeframe that encompasses major global milestones, including the transition to the SDGs and the FP2030 global partnership. The FP2030 partnership set ambitious targets to expand access to voluntary family planning for 120 million additional women and girls by 2030, with a particular focus on low- and middle-income countries, including SSA. Despite these commitments, adolescents in SSA continue to face disproportionately high unmet contraceptive needs, with adolescent birth rates remaining among the highest in the world. This context directly motivates the need for this scoping review to understand what specific barriers persist despite these high-level policy commitments, and to identify what targeted interventions are still needed to translate global goals into improved outcomes for adolescents in SSA.

## Materials and methods

Given the broad and exploratory nature of the research question, to identify and document barriers to accessing contraceptive services for adolescents in SSA, and to provide evidence-based recommendations to address such barriers, a scoping review methodology was selected [14]. This approach is suitable for mapping existing evidence, identifying key concepts, and highlighting gaps in the literature. The review followed the Arksey and O’Malley five-stage framework, which includes: (1) identifying the research question, (2) identifying relevant studies, (3) study selection, (4) charting the data, and (5) collating, summarising, and reporting the results. Additionally, the methodology was enhanced with the Joanna Briggs Institute guidelines [15]. These frameworks were adopted to ensure a systematic and transparent approach to the synthesis.

### Research question

The scoping review was guided by the question: *‘What are the barriers to accessing contraceptive services for adolescents in Sub-Saharan Africa?’* and was derived using the PICO framework for the scoping review, where P (Population), I (Intervention), C (Comparison) and O (Outcome) were the parameters. In this regard, the research question was structured as follows:

P (Population) = Adolescents aged 10–19 years

I (Intervention) = Contraceptive Services

C (comparison) = No direct comparison

O (Outcome) = Documented outcome that identifies barriers to accessing contraceptive services in SSA, and how these barriers can be addressed to improve access.

### Eligibility criteria

The PRISMA-ScR (Preferred Reporting Items for Systematic Reviews and Meta-Analyses extension for Scoping Reviews) was used to guide the transparent reporting of the selection and screening process. This reporting framework enhances the clarity, transparency, and consistency in documenting how studies were identified, screened, included, or excluded based on pre-determined eligibility criteria appropriate for the scoping review methodology.

#### Inclusion criteria

1. Peer-reviewed journal articles, reports, and grey literature examining barriers to adolescent contraceptive access, aged 10–19 years
2. Full-text articles
3. Studies published from January 2010 to January 2025
4. Studies conducted within SSA
5. Qualitative, quantitative, and mixed-methods studies
6. Studies focusing on any contraceptive method (e.g. condoms, oral contraceptives, injectables, and long-acting reversible contraceptives)
7. Both published and unpublished articles written in English

#### Exclusion criteria

Studies were excluded if they lacked empirical data (e.g. editorials or commentaries); had unclear or poorly described methodologies (for example, studies that did not specify their study design, data collection methods, or analytical approach, making quality appraisal and interpretation impossible); or if their primary focus was on contraceptive effectiveness rather than barriers to access.

### Search strategy

A comprehensive literature search was conducted across Google Scholar, PubMed, Scopus, Medline, Web of Science, Embase, AJOL, and JSTOR. The electronic search utilised MeSH terms and keywords organised into three main categories: Population (e.g. ‘adolescent,’ ‘teenager’), Concept (e.g. ‘contraceptive services,’ ‘barriers’), and Setting (e.g. ‘Sub-Saharan Africa,’ specific country names). A manual search was also performed via ‘snowballing,’ which involved reviewing the reference lists of included articles and utilising the ‘related articles’ feature in electronic databases. Grey literature was retrieved from the repositories of major global health organisations, including the WHO, the United Nations Population Fund, and the Guttmacher Institute.

#### Study selection and screening

Articles retrieved from both electronic and manual searches were exported into the Mendeley reference management tool, and duplicates were removed. To ensure reliability, a two-stage screening process was established to determine the eligibility of the retrieved data. The first phase of the process involved a quick independent assessment of the title and abstract of each respective record against the pre-established eligibility criteria by two reviewers. Studies that do not meet the eligibility criteria were outrightly eliminated. In the second phase of the screening process, however, full-text reviews were conducted for all articles meeting the title and abstract screening criteria. Any disagreements between the two primary reviewers regarding inclusion were resolved through consultation with a third reviewer to reach a consensus.

#### Data extraction tool and process

Two data extractors extracted the data into a standardised Microsoft Excel template, capturing descriptive indicators (author, year, country, objective), methodological variables (design, population), and main findings of the studies.

### Data synthesis and analysis

Data extracted from the included studies were analysed using thematic synthesis to map the range and nature of reported barriers. Findings were organised using the Socio-Ecological Model (SEM), which categorises barriers across individual, sociocultural, health system, and legal and policy levels. The framework was applied to structure and classify evidence rather than to test theoretical relationships.

A qualitative coding process was undertaken to identify recurring concepts reported across studies. Two reviewers independently coded the extracted data, compared emerging codes, and resolved discrepancies through discussion to ensure consistency. Following agreement on a coding framework, related codes were grouped into broader categories and mapped to the relevant SEM domains. These categories were iteratively reviewed and refined to describe overarching patterns in the evidence. An audit trail documenting coding decisions and framework development was maintained to enhance transparency and reproducibility. Findings from the thematic synthesis were presented narratively to describe the distribution and characteristics of identified barriers.

However, descriptive analysis in the form of percentages and frequencies was performed on the descriptive information (year of publication, study design, country) of the included studies to identify the general pattern and distribution of the studies.

#### Ethical considerations

Although the protocol was not registered, ethical approval was obtained from the MSc Research Ethics Committee of the London School of Hygiene and Tropical Medicine with number [Ref: 31,740]. Additionally, the review strictly adhered to all pre-defined internal protocols to ensure rigour.

### Limitations of the review

The review was limited to only studies conducted and published in English. This language constraint had the potential to eliminate pertinent studies that were conducted and published in languages other than English. Besides that, as in other reviews, there is a high possibility of several unpublished studies not included in the review, known as publication bias. This may have affected the findings of this study. Additionally, this scoping review intentionally included existing systematic reviews alongside primary studies. This ‘review of reviews’ approach was chosen to capture broad, synthesised regional evidence and to identify where higher-level evidence already exists versus where primary research is still lacking. We acknowledge that this may introduce some data overlap where primary studies are cited both directly and within the included reviews; however, this approach was deemed necessary to provide the most comprehensive regional overview possible.

## Results

**Figure 1.**
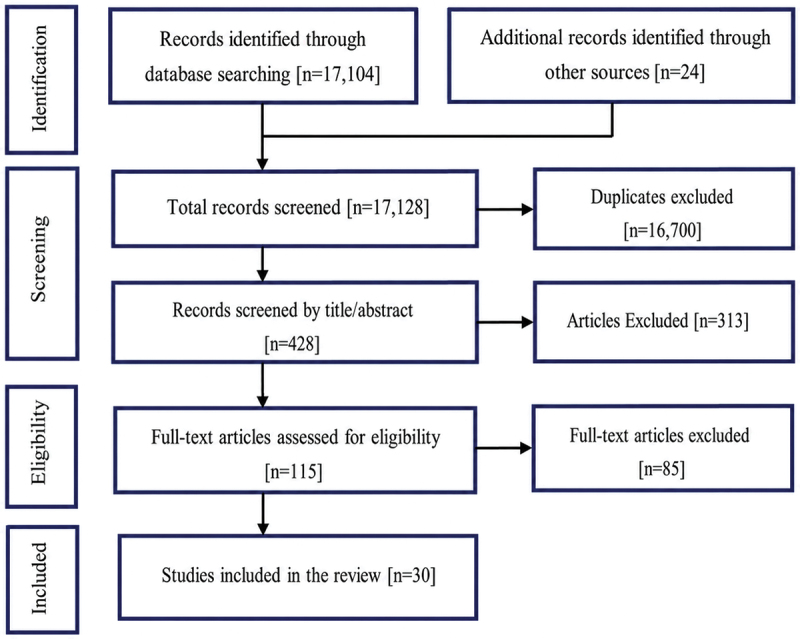
Flow diagram of the studies included in the review.

A total of 17,128 articles were retrieved via database searching and other sources, such as manual searching by using different search terms. The electronic search yielded 17,104 articles. Of the 17,128 articles retrieved, 428 of them were eligible for title and abstract evaluation. Subsequently, the title and abstract evaluation excluded 313 of the 428 studies. Hence, a total of 115 articles were taken through full-text assessment, which saw 85 of the articles eliminated due to varied reasons (articles older than the study duration, conducted outside the defined study area, didn’t report on the outcome variable); hence, 30 articles were included for further analysis. This is shown in Figure 1.

### Study characteristics

**Figure 2.**
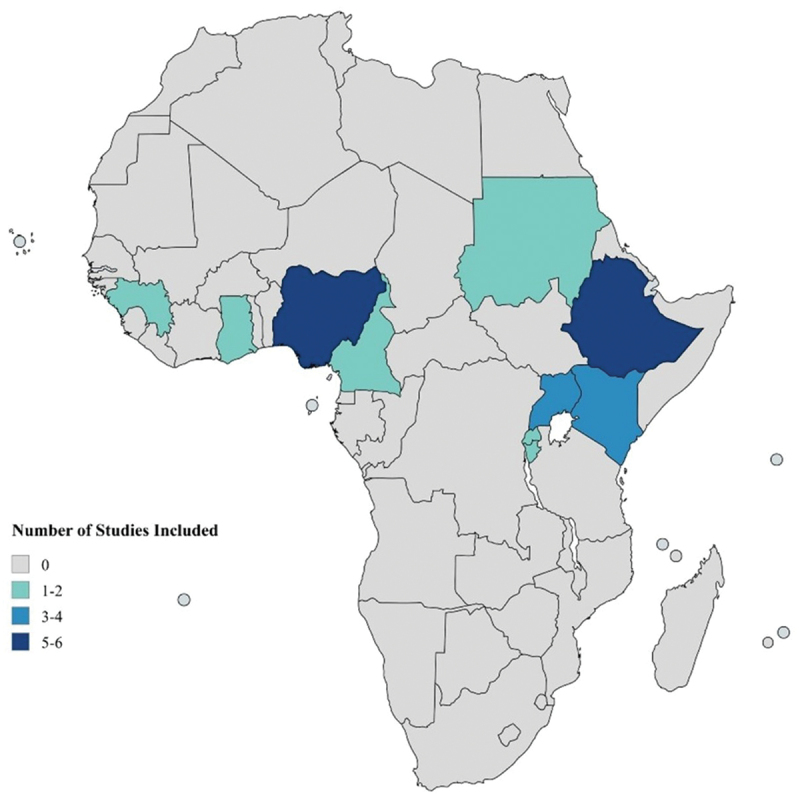
Geographical distribution of studies in the review.

After a rigorous selection process and descriptive analysis, 30 relevant studies were retained for the review. The analysis revealed that 13 [43%] of the studies were qualitative studies, while 8 [27%] were reviews. Cross-sectional studies accounted for 5 [17%] of the overall studies, compared to mixed-methods studies, which accounted for 3 [10%] of the included studies. Again, descriptive analysis of the geographical location of the various studies showed that 8/30 [26.7%] were reviews conducted in SSA, while 5/30 [17%] were conducted in Nigeria and Ethiopia, respectively. The studies included in the review had 4/30 [13%] from Uganda compared to 3/30 [10%] from Kenya. Ghana and Rwanda, however, accounted for 7% of each of the included studies. Guinea, Sudan, South Africa, and Cameroon each had 3% representation in the included studies for this review. This breakdown is illustrated in Figure 2.

### Identified gaps in the literature

Although the included studies provided valuable insights into the barriers to adolescent contraceptive access in SSA, several important gaps were identified.

Only three out of the 30 studies [10%] specifically addressed the experiences of marginalised adolescent populations, such as adolescents with disabilities, LGBTQ+ youth, or those living with HIV. The majority of studies focused on general adolescent populations, limiting understanding of how intersecting vulnerabilities shape access to contraception.

In terms of geographic representation, West Africa accounted for the largest proportion of studies 12/30 [40%], followed by East Africa 9/30 [30%]. Southern Africa and Central Africa were underrepresented, contributing only five [17%] and four studies [13%], respectively. This uneven distribution limits our ability to draw regional comparisons and may overlook country-specific barriers.

Regarding study design, the vast majority of articles 26/30 [87%] were cross-sectional, with only two longitudinal studies and two program evaluations, suggesting a limited understanding of how barriers change over time or in response to policy shifts. Moreover, only four studies [13%] examined the effectiveness of interventions aimed at improving contraceptive access among adolescents.

Finally, only five studies [1.7%] included the perspectives of key influencers, such as healthcare providers, parents, or community leaders, even though these actors play a critical role in enabling or hindering access to services. These gaps highlight the need for more inclusive, regionally diverse, and implementation-focused research to support the design of responsive policies and programs tailored to the needs of all adolescents across SSA.

## Discussion

### Overview of themes

This review synthesised evidence on barriers to adolescent access to contraceptive services across SSA and revealed five dominant themes: individual-level barriers, sociocultural factors, economic factors, health system barriers, and policy and legal restrictions. Sociocultural barriers to contraceptive access include stigma, religious beliefs, and parental disapproval. Economic barriers, such as the cost of contraceptives and transportation, further limit access. Health system factors, including negative provider attitudes and concerns about confidentiality, also deter adolescents from accessing contraceptive services. Additionally, age restrictions and consent requirements constitute policy and legal barriers to accessing contraceptive services among adolescents in SSA.

Although these barriers were widely reported, their expression varied across countries and regions, including West, East, and Central Africa, highlighting the importance of context-specific interpretation and locally tailored interventions.

Across sub-regions, distinct patterns in the dominant barriers emerged. In West Africa (e.g. Nigeria, Ghana), individual knowledge gaps, parental control, stigma, and negative provider interactions appear particularly prominent. In East Africa (e.g. Ethiopia, Uganda), barriers are more strongly shaped by cultural prohibitions around premarital sexuality, structural constraints on access to accurate information, and limited availability of youth-friendly services. In Southern Africa (e.g. South Africa), provider stigma and concerns around confidentiality are more frequently emphasised. In Central Africa (e.g. Cameroon), institutional religious opposition appears to play a more visible role in shaping contraceptive attitudes. In addition, rural settings across SSA tend to experience more pronounced economic and geographic barriers, including distance to facilities and indirect costs, whereas urban settings are more commonly characterised by service-level constraints such as provider attitudes, long waiting times, and limited privacy. These patterns should be interpreted cautiously given variability in study distribution across regions, but they provide a useful basis for context-sensitive interpretation.

### Individual barriers

Limited knowledge and persistent misconceptions regarding contraceptive services were consistently reported across SSA, though their specific manifestations varied [16–18]. In West Africa, particularly in Nigeria, evidence emphasised inadequate reproductive health literacy and misinformation about contraceptive use as primary determinants of low uptake [11]. Adolescents in these settings frequently lacked basic knowledge of available methods and service points.

In contrast, studies from East Africa, including Ethiopia and Uganda, highlighted structural constraints on access to accurate information, including limited counselling services and inadequate youth-friendly information channels [16–18]. Here, the barrier was less a complete absence of awareness and more restricted access to reliable guidance and supportive services.

Evidence from Ghana and other West African settings also showed that misconceptions, particularly fears of infertility and adverse effects, were reinforced through peer networks and community narratives [19]. These findings suggest potential regional variations in informational barriers: while foundational knowledge gaps predominate in some settings, fear-driven misconceptions and misinformation networks are more influential in others.

Although some included studies referenced unsafe abortion practices as downstream consequences of limited contraceptive access, unsafe abortion was not a primary outcome of this review [20,21]. Only a minority of included studies reported abortion-related practices, and these findings were interpreted as contextual evidence illustrating potential consequences of limited access rather than as a central focus of analysis.

The diversity of knowledge barriers across regions suggests that comprehensive sexuality education must be adapted to local informational needs, addressing basic reproductive health literacy in some contexts while focusing on myth correction and counselling access in others.

### Sociocultural barriers

Sociocultural norms were a pervasive constraint across SSA, but their mechanisms and intensity varied substantially across settings [16,22–24]. In East Africa, particularly in Ethiopia, strong cultural prohibitions against contraceptive use among unmarried adolescents were reported, reflecting deeply embedded norms surrounding premarital sexual behaviour [16,22]. These norms often operated through community surveillance and social sanctions.

In West Africa, including Nigeria and Ghana, sociocultural barriers are frequently manifested through parental control, family disapproval, and stigma associated with adolescent contraceptive use [11,25]. Family gatekeeping and religious expectations were particularly influential in shaping adolescents’ health-seeking behaviour in these contexts.

Evidence from Central Africa, including Cameroon, highlighted religious opposition as a central determinant of contraceptive attitudes among adolescents [26], reflecting the role of institutional religious structures in shaping reproductive norms across the sub-region. Across regions, peer influence also contributed to contraceptive perceptions, though its role varied by context. In urban settings, peer networks and social interactions appeared more influential, whereas in rural contexts, family authority and community norms were more dominant [25,27]. These variations indicate that sociocultural barriers operate through different social structures across settings, requiring context-sensitive interventions that engage the most influential actors within each community.

### Economic barriers

Economic barriers emerged as a major determinant of access across SSA, particularly service costs and transportation expenses [28–31]. Evidence from Uganda demonstrated that transportation challenges and service fees significantly constrained access, particularly for rural adolescents [29]. Similar financial constraints were reported in multi-country studies involving Nigeria, Kenya, and Guinea [28,30,31]. This indicates consistent cost-related barriers across both East and West African settings.

However, economic barriers were shaped by local health system structures and socioeconomic conditions. In resource-constrained rural settings, indirect costs, including travel time and lost income, appeared particularly restrictive, whereas in urban settings, service fees were more frequently cited as barriers [10,11,32,33].

The review also identified limited evidence on cost differences across contraceptive methods. Few studies distinguished between long-acting reversible contraceptives (LARCs) and short-acting methods. Where evidence was available, higher initial costs and provider requirements appeared to constrain LARC uptake, while recurrent costs influenced short-acting method use [10,34]. However, inconsistent measurement across studies prevents definitive conclusions. This gap highlights the need for method-specific economic analyses to guide targeted financing strategies.

### Health system barriers

Health system constraints were widely reported across SSA, extended beyond individual provider behaviour to the broader service environment, though their nature differed by country context [12,23,25,31,35]. In Nigeria, provider attitudes and restrictive service hours were key barriers, while studies in Guinea identified structural challenges such as contraceptive stockouts, geographic inaccessibility, and limited provider training [31,36]. Evidence from Ghana similarly highlighted negative provider interactions and a lack of privacy protections as major deterrents [25].

Across East African contexts, barriers often reflected limited availability of youth-friendly services and inadequate provider capacity, whereas in some South African settings, such as South Africa and Botswana, provider stigma and confidentiality concerns were more prominent [37,38]. These regional differences suggest that improving access requires both structural system strengthening and behavioural interventions targeting provider practices.

Notably, these health system barriers interact with contextual factors, with rural settings more affected by infrastructure and access limitations, while urban settings more frequently report service quality and provider-related concerns, further reinforcing the need for context-sensitive service delivery strategies.

### Policy and legal barriers

Policy and legal restrictions also shaped access to contraceptive services across SSA. Studies reported restrictive eligibility criteria, parental consent requirements, and weak adolescent-focused policy frameworks as significant barriers [27,39–41]. Evidence from Kenya demonstrated that rigid consent requirements limited adolescent access to reproductive health services [39]. Even where national policies formally supported adolescent access, as in Ethiopia and Rwanda, facility-level implementation was inconsistent, creating de facto barriers [42–45]. More broadly, legal ambiguity often enabled discretionary gatekeeping by providers. Policy analyses across multiple SSA settings revealed fragmented institutional coordination and limited integration of adolescent needs within national reproductive health strategies [27]. This observation contributes to inconsistent messaging and service fragmentation, hence highlighting the gap between policy intent and operational reality.

Variation in policy environments across countries highlights the importance of national-level governance structures in shaping access, suggesting that policy reform must be tailored to country-specific institutional contexts.

Importantly, the effects of these policy and legal barriers appear to vary by context, with evidence from selected settings suggesting that consent requirements and implementation gaps may be more restrictive in certain health system and sociocultural environments than others.

Nonetheless, few studies directly examined legal texts or policy enforcement mechanisms, limiting definitive conclusions regarding legal causality. Although coordination challenges between the health and education sectors were mentioned, they were systematically evaluated. As such, conclusions regarding intersectoral fragmentation remain interpretive rather than empirically robust.

### Recommendations

#### Further studies

It is worth noting that current evidence on barriers to adolescents’ access to contraceptive services is largely focused on heterosexual adolescents, leaving a gap in understanding the barriers to accessing contraceptive services among marginalised adolescents, such as those with disabilities, LGBTQ+ youth, whose barriers may significantly differ from those of their peers. Hence, it is imperative to explore the barriers in accessing contraceptives among these vulnerable populations with unique challenges.

Additionally, further studies can explore similar themes with the inclusion of languages such as French, Arabic, Portuguese, and German, which will provide a general perspective of the diversity in the region.

Furthermore, measurement heterogeneity across studies further limits cross-country comparisons; hence, future research should prioritise disaggregated analyses based on geography.

### Policy actions to improve adolescent access to contraceptive services

To address the identified barriers in a structured and context-sensitive manner, policy actions have been aligned with the five domains of the Socio-Ecological Model (SEM), ensuring that recommendations correspond directly to the levels at which barriers operate. Where appropriate, distinctions are made between rural and urban settings and across sub-regions.

1. **Individual Level:**
  - Strengthen comprehensive sexuality education and reproductive health literacy programmes tailored to local knowledge gaps.
  - In West African contexts, prioritise foundational reproductive health knowledge, while in settings where awareness exists but misinformation persists, emphasise myth correction and counselling services.
2. **Interpersonal/Sociocultural Level:**
  - Promote community engagement initiatives involving parents, religious leaders, and peer networks to reduce stigma and shift norms.
  - Interventions should be adapted to context: for example, engaging family gatekeepers in West Africa, addressing community surveillance and premarital norms in East Africa, and working with institutional religious actors in Central Africa.
3. **Community/Environmental Level:**
  - Improve service accessibility through outreach and community-based delivery models.
  - In rural settings, prioritise mobile outreach and decentralised distribution to address distance and cost barriers; in urban settings, focus on extended service hours, reduced waiting times, and privacy-enhancing facility designs.
4. **Health System Level:**
  - Strengthen adolescent-friendly service provision through provider training and supportive supervision.
  - Address stockouts, improve service availability, and enhance confidentiality protections.
  - Interventions should reflect context-specific needs, with greater emphasis on infrastructure and workforce capacity in rural areas and service quality and provider attitudes in urban settings.
5. **Policy/Legal Level:**
  - Review and, where appropriate, revise parental consent requirements to improve adolescent access to services.
  - Expand financial protection mechanisms to reduce cost barriers.
  - Strengthen coordination between health, education, and legal sectors and integrate adolescent reproductive health into national strategies.
  - These policy actions should be adapted to national legal and institutional contexts, recognising variation in implementation capacity and governance structures across SSA.
6. **Impact and Evaluation:**
  - Establish monitoring frameworks to assess the effectiveness of interventions.
  - Incorporate adolescent feedback into service design and policy refinement to ensure responsiveness to user needs.

**Figure:**
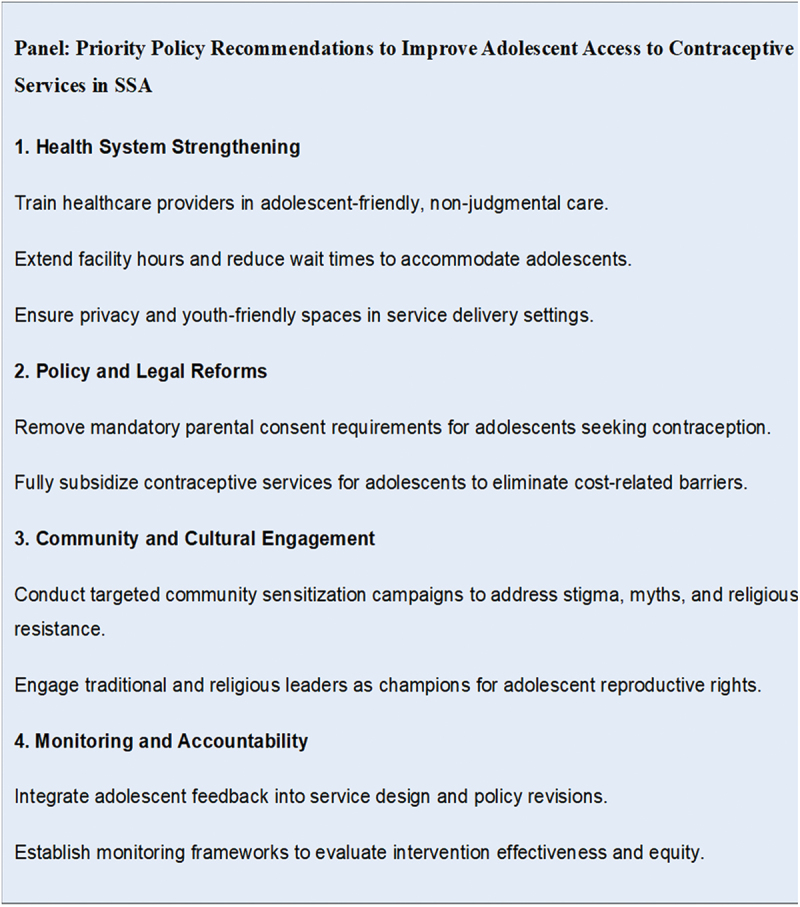

## Conclusion

Overall, this review demonstrates that barriers to adolescent contraceptive access in SSA are multifaceted but highly context-dependent. While knowledge deficits, sociocultural norms, economic constraints, health-system limitations, and policy restrictions are widely observed, their underlying drivers and relative importance differ across West, East, and Central African settings. Effective interventions must therefore be contextually grounded, reflecting local sociocultural dynamics, health system capacity, and policy environments rather than relying on uniform regional approaches.

## Supplementary Material

PRISMAScRFilled.pdf

## Data Availability

The data supporting the findings of this scoping review were extracted from publicly available studies. The extracted and charted dataset used for analysis is available from the corresponding author upon reasonable request.

## Preprints

This manuscript has not been posted on a preprint server.
